# Resonant light scattering from a single dielectric nano-antenna formed by electron beam-induced deposition

**DOI:** 10.1038/srep10400

**Published:** 2015-05-19

**Authors:** Eun-Khwang Lee, Jung-Hwan Song, Kwang-Yong Jeong, Ju-Hyung Kang, Hong-Gyu Park, Min-Kyo Seo

**Affiliations:** 1 Department of Physics and Institute for the NanoCentury, KAIST, Daejeon 305-701, Republic of Korea; 2 Department of Physics, Korea University, Seoul 136-701, Republic of Korea

## Abstract

Dielectric nano-antennas are promising elements in nanophotonics due to their low material loss and strong leaky-mode optical resonances. In particular, light scattering can be easily manipulated using dielectric nano-antennas. To take full advantage of dielectric nano-antennas and explore their new optical applications, it is necessary to fabricate three-dimensional nano-structures under arbitrary conditions such as in non-planar substrates. Here, we demonstrate full-visible-range resonant light scattering from a single dielectric optical nano-rod antenna. The nano-rod antenna was formed by electron beam-induced deposition (EBID), a promising three-dimensional nanofabrication technique with a high spatial resolution. The nano-rods consist of amorphous alloys of C and O, with a width of 180 nm on average and a length of 4.5 μm. Polarization-resolved dark-field scattering measurements show that both transverse-electric and transverse-magnetic mode resonances cover the full visible range as the height of the nano-rod antenna varies from 90 to 280 nm. Numerical simulations successfully reproduce the measured scattering features and characterize the modal properties, using the critical points dispersive dielectric constant of the EBID carbonaceous material. Our deep understanding of resonant light scattering in the EBID dielectric nano-antenna will be useful for near-field measurement or for the implementation of three-dimensional nanophotonic devices.

Optical resonances of dielectric or metallic nano-structures can support large scattering/absorption cross-sections and interface free-propagating light with electromagnetic fields localized in the sub-wavelength area. For the last decades, plasmonic metal nano-antennas that provide extreme field concentration and strong light-matter interaction have been intensively investigated and applied to fluorescence spectroscopy^1^,^2^,^3^,^4^,^5^, Raman spectroscopy^6^,^7^,^8^,^9^,^10^, nonlinear optics^11^,^12^, and near-field microscopy^13^,^14^. More recently, dielectric nano-structures have also received attention due not only to their low material losses but also to their leaky-mode resonances. The leaky-mode resonances, termed Mie resonances, overcome the optical diffraction limit in dielectric materials and achieve strong light scattering and absorption for photodetectors^15^,^16^, solar cells^17^,^18^, nanolasers^19^, and light emitting diodes^20^. In particular, since dielectric nano-antennas support both electric and magnetic mode resonances at the same time and combine them in simple geometries, forward and backward scattering can be easily manipulated^21^,^22^,^23^,^24^.

To extend the utility of optical nano-antennas and explore their new applications, it is necessary to fabricate on-demand three-dimensional (3D) nano-structures, with arbitrary shape and substrate. Conventional top-down fabrication techniques such as electron-beam lithography are not suitable to form 3D nano-antennas on a bumpy or structured substrate (e.g., near-field scanning optical microscope (NSOM) tip). Although two-photon polymerization can produce arbitrary structures^25^,^26^, the size of the product is typically larger than the micro-scale, the choice of materials is limited, and the post-process to remove un-polymerized materials is required. Recently, smaller features sizes of a few tens of nanometres have been demonstrated using multi-photon polymerization and stimulated emission depletion direct writing techniques^27^,^28^. Also, a large range of materials is accessible in combination with template inversion schemes^29^. On the other hand, electron beam-induced deposition (EBID), which uses a scanning electron microscope (SEM) or a transmission electron microscope (TEM), is a promising technique to precisely fabricate complex 3D nano-structures at any desired position on the substrate or even in a free-standing geometry^30^,^31^. In addition, one can deposit metallic or dielectric materials using the EBID technique by choosing the proper precursor gas. Recently, EBID nano-structures have been used to improve the performance of nanophotonic devices and measurement techniques including photonic crystal lasers^32^,^33^, tip-enhanced Raman spectroscopy (TERS)^34^, and so on. However, there are limited studies on the applications of EBID nano-structures as optical nano-antennas and the characterization of their modal properties.

In this study, we demonstrated a single EBID dielectric nano-rod antenna, and measured the resonant scattering images and spectra that depend on the height of nano-rod. In the transverse-electric (TE) and transverse-magnetic (TM) resonances, tunable structural colour generation was observed over the whole visible spectrum. Numerical simulations successfully reproduced the experimental results and characterized the modal properties of the EBID nano-rod antenna.

## Results

### Fabrication of EBID nano-rod antennas

**Figure 1:**
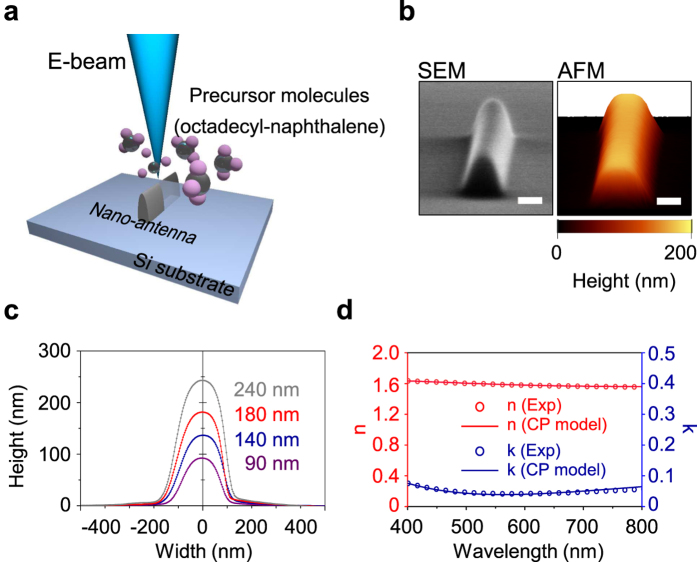
EBID dielectric nano-rod antenna. (**a**) Schematic of the EBID procedure used to form a 3D nano-rod antenna. Vaporized oil molecules (octadecyl-naphthalene) from the diffusion pump function as precursor gas. (**b**) SEM (left) and AFM (right) images of a fabricated EBID carbonaceous dielectric nano-rod antenna. The height, width, and length are 170 nm, 170 nm, and ~4.5 μm, respectively. The scale bars are 100 nm. (**c**) Cross-sectional views of the selected nano-rod antennas measured by AFM. The height changes from 90 to 240 nm. (**d**) Measured refractive index (left axis; red circles) and extinction coefficient (right axis; blue circles) of an EBID block, using spectroscopic ellipsometry (see Supplementary Figure S2). The block was fabricated under the same conditions as the EBID dielectric nano-rod antenna at a current of ~130 pA. The experimental data were fitted to the CP model (red and blue solid curves).

To form EBID nano-rod antennas on the silicon substrate, the electron beam is focused and scanned along a straight line. Vaporized oil molecules from the diffusion pump in the SEM (Hitachi S-2500) function as precursor gas. The precursor molecules are chemically transformed by the highly focused electron beam, and are dissociated into target deposit and volatile by-product^35^. Only target deposit adsorbs onto a substrate (Fig. 1a). The structural parameters of the nano-rods are easily controlled using the dose of the electron beam. Figure 1b shows SEM and atomic force microscopy (AFM) images of the fabricated EBID nano-rod antenna. The length of the nano-rods is fixed at ~4.5 μm and the heights range from ~90 to ~280 nm. The width of the nano-rods is less sensitive to the dose of the electron beam, compared to the height, and varies from ~170 to ~210 nm (Fig. 1c). The AFM image in Fig. 1b shows that the surface roughness of the nano-rod is only ~1.5 nm. This indicates that 3D nano-structures with smooth surfaces can be easily demonstrated by EBID with simple fabrication procedure, while they would be difficult to fabricate using conventional top-down lithography techniques. Additionally, energy-dispersive X-ray spectroscopy (EDS) measurements show that the EBID nano-rods are amorphous alloys of C and O. These alloys have a C:O atomic ratio of ~88:12. This ratio is almost independent of the electron beam current (see Supplementary 1).

To investigate the wavelength-dependent refractive index and extinction coefficient of the EBID carbonaceous material, we performed spectroscopic ellipsometry measurements (Fig. 1d; see also Supplementary Figure S2). Since the nano-rod is too small for us to be able to obtain the refractive index directly, we analysed the reflectivity spectrum in a block with a size of 73 × 78 μm^2^, which was fabricated using the same EBID procedure as the nano-rod (see Methods). The ellipsometry measurement shows that the refractive index of the block gradually changes from 1.63 to 1.56 and the extinction coefficient is ~0.05 on average, as the wavelength increases from 400 to 800 nm (Fig. 1d). This informs us that our EBID structure consists of a dielectric carbonaceous material. We also examined the EBID block fabricated with an electron beam current of ~830 pA, and compared it with the block fabricated with a current of ~130 pA. The refractive indices and material composition of both blocks are almost identical (see Supplementary Figure S2), and thus the material properties of the EBID structures are independent of the EBID fabrication conditions. In addition, the consistency between the real refractive index and the imaginary extinction coefficient was verified using the Kramers-Kronig relations. We fit the measured indices of the EBID carbonaceous material using the critical points (CP) model^36^,^37^, which will be used for finite-difference time-domain (FDTD) simulations (See Supplementary Table S1).

### Measurement of scattering images

**Figure 2:**
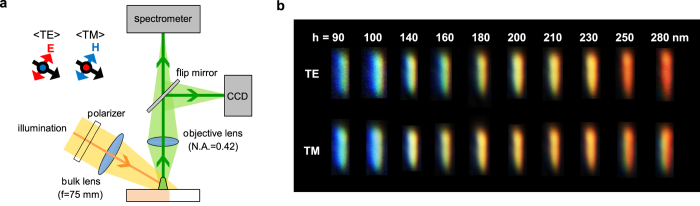
Full-visible range light scattering of single EBID dielectric nano-rod antennas. (**a**) Schematic of the scattering measurement set-up based on dark-field microscopy. Linearly polarized illumination by a tungsten-halogen lamp is incident on the single nano-rod antenna. The scattered light is collected normal to the substrate using a 50× long-working-distance microscope objective lens (numerical aperture of 0.42) and fed into either a spectrometer or a colour CCD camera. (**b**) Measured dark-field optical images of nano-rod antennas for TE- (upper) and TM-polarized (lower) illumination. As the height of the nano-antennas changes from 90 (left) to 280 nm (right), vivid structural colours are generated from blue to green yellow and red for both TE and TM polarizations.

To measure the scattering properties of the fabricated EBID nano-rod antennas, we used a home-built dark-field microscopy set-up^38^ (Fig. 2a). White light from the broadband tungsten-halogen lamp is obliquely incident on the nano-rod antennas at an angle of ~60° with respect to the substrate. A bulk lens with a focal length of 75 mm and a linear polarizer are located in front of the illumination lamp. The incident light is separated into transverse-electric (TE) and transverse-magnetic (TM) polarization states, which are perpendicular and parallel to the direction of the nano-rod length, respectively. Then, the scattered light from the nano-rod antenna is collected using a 50× long-working-distance microscope objective lens with a numerical aperture of 0.42. The scattered light is then fed into either a spectrometer (SpectraPro monochromator combined with Pixis array detector, Princeton Instruments) or a colour charge-coupled device (CCD) camera.

The measurements show that the dielectric nano-rod antenna supports resonant light scattering over the entire visible wavelength range depending on its height. Dark-field scattering images of the single nano-rod antennas for both TE and TM polarizations were captured using the colour CCD camera (Fig. 2b). As the height of the nano-antennas changes from 90 to 280 nm, the colour of the scattered light continuously changes from blue to green, yellow and red. The TE polarization shows a better colour gamut, particularly in green and red, than the TM polarization. This is because the TE-polarized incidence supports a scattering spectrum with a narrower bandwidth and a more distinct resonance peak than the TM-polarized incidence. On the other hand, the scattering intensity of the TM polarization is stronger than the TE polarization. The details of the scattering intensity and its spectrum will be discussed in the following section. We note that such vivid colour generation covering red, green, and blue with a sub-wavelength footprint makes the nano-rod antenna a promising candidate for achieving high-definition colour units for display or ornaments that break the diffraction limit of light.

### Measurement and simulation of scattering spectra

**Figure 3:**
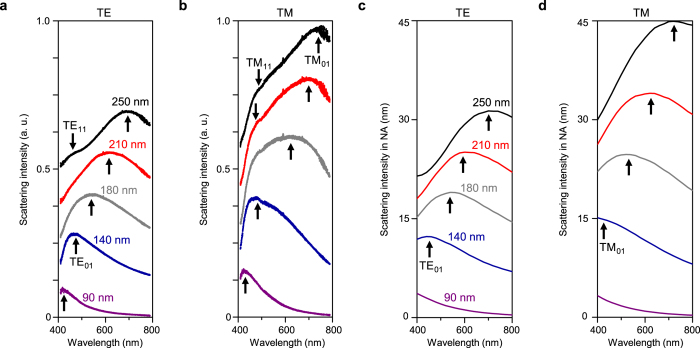
Measured and simulated scattering spectra. (**a**,**b**) Measured dark-field scattering spectra of EBID nano-rod antennas with different heights for TE (**a**) and TM (**b**) polarizations. The heights vary from 90 to 250 nm. The scattering intensity (*y*-axis) was obtained by taking the ratio of the intensity of the collected scattering signal to that of the incident illumination. (**c**,**d**) Simulated scattering spectra of the nano-rod antennas with the same heights as the experiment. 2D FDTD simulations were performed for TE (**c**) and TM (**d**) polarizations.

We analysed the scattering properties of the EBID nano-rod antenna more quantitatively by measuring the polarization-resolved dark-field scattering spectrum. In the TE and TM scattering spectra of selected nano-rod antennas, the collected scattering intensity normalized by the incident illumination intensity was plotted as a function of the wavelength (Fig. 3a,b). The spectra reveal several key features which are as follows. First, the distinctive resonance peaks agree well with the colours of the dark-field images in Fig. 2b. Second, the shift of the peaks to longer wavelengths is clearly observed as the height of the nano-rod antenna increases, as is also shown in Fig. 2b. Third, the TM scattering exhibits stronger intensity and a longer resonance wavelength than the TE scattering. Fourth, the resonant peaks can be identified as the TE_01_ and TE_11_ modes (in the TE scattering) and as the TM_01_ and TM_11_ modes (in the TM scattering). The notation TE_*ml*_ or TM_*ml*_ specifies the azimuthal mode number, *m,* and the radial order number, *l,* based on the electric field distribution. In particular, the nano-rod antenna with a height of ~250 nm supports the TM_01_ mode resonance at a wavelength of 740 nm. The TM_01_ mode resonance shows a large wavelength shift of ~315 nm, which spans the entire visible wavelength range as the height increases from 90 to 250 nm. As the height increases, the TE_01_ mode resonance shifts from ~420 to ~700 nm, also covering the visible wavelength range. When the height of the nano-rod antenna is larger than 200 nm, the higher-order TM_11_ and TE_11_ mode resonances also appear in the visible wavelength range.

We compared the experimentally measured spectra with two-dimensional (2D) FDTD simulation results (Fig. 3c,d). The length of the nano-rod antenna is long enough that we could carry out 2D simulations in the spectral range from 400 to 800 nm. The monochromatic plane wave is injected at an angle of 60° with respect to the substrate. Then, we plot the light intensity scattered toward the vertical direction with a divergence angle of ±25°, corresponding to the numerical aperture of the microscope objective used in experiment, as a function of the incident wavelength (see Methods). To reflect the cross-section morphology of the actually fabricated nano-rods, we used an isosceles trapezium in combination with a half circle on top (see Methods and Figure S3a). The calculated scattering intensity spectra successfully reproduced the experimental data, such as the resonance wavelength, scattering intensity, and the wavelength shift depending on the height of the nano-antenna. We also observed that the TM_01_ mode resonance has a longer wavelength and stronger scattering intensity as compared to the TE_01_ mode resonance, which is in good agreement with the experiment. We note that the intensity ratio between the TE and TM polarizations in the simulation is similar to the measured one, but the higher-order resonance observed in the measurement does not appear in the simulation. Since the higher-order modes are more sensitive to the incident or collection angles compared to the lower-order ones, it is challenging to find out all resonant modes in the simulation.

### Characterization of mode properties

**Figure 4:**
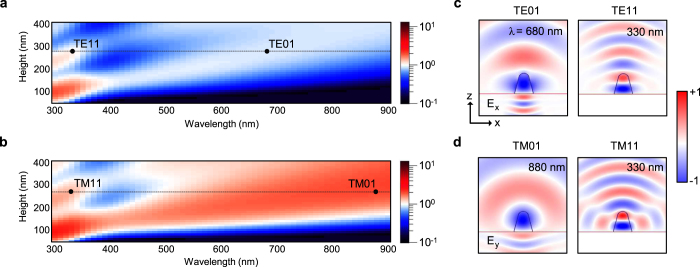
Calculated resonant modes of the EBID dielectric nano-rod antenna. (**a**,**b**) 2D maps of the total scattering efficiency on a log scale as a function of the wavelength and the height of the nano-rod antenna for TE- (**a**) and TM-polarized (**b**) illumination. (**c**) E_x_-field profiles of the TE_01_ (*λ* = 680 nm) and TE_11_ modes (*λ* = 330 nm) for the nano-rod with a height and width of 280 and 178 nm, respectively. (**d**) E_y_-field profiles of the TM_01_ (*λ* = 880 nm) and TM_11_ modes (*λ* = 330 nm) of the nano-rod shown in (**c**). The field components in the profiles are those of the scattered field, not the total field.

For a better understanding of the resonant modes, we calculated 2D maps of the total scattering efficiency as a function of the incident wavelength and height of the nano-rod antenna, for the TE and TM polarizations (Fig. 4a,b). Here, the total scattering efficiency is defined as the ratio of the total scattering cross-section to the height of the nano-rod antenna. The total field/scattered field (TFSF) method was used to calculate the total scattering cross-section including light scattering to air and substrate from a normally incident plane wave (see Methods). The FDTD result shows that the peak wavelength of the scattering efficiency is linearly red-shifted as the height of the nano-rod increases. As observed in Fig. 3c,d, the TM_01_ mode appears at a longer wavelength than the TE_01_ mode because the TM_01_ mode has larger effective index. Multiple scattering resonances also appear at a height of >240 nm. We note that the dielectric EBID nano-rod antenna supports resonant light scattering for which the cross-section is larger than the physical cross-section for the TM polarization. In addition, we calculated the absorption and extinction efficiencies of the nano-rod antenna (Supplementary Figure S4a-b). The absorption efficiency is non-negligible due to a relatively high extinction coefficient of the deposited material (Fig. 1d), and also shows the resonance features similar to the total scattering efficiency of Fig. 4a,b.

Figure 4c,d show the TE- and TM-polarized electric-field distributions of the scattered field. In these field profiles, the azimuthal mode number, *m*, is clearly observed, which corresponds to the number of nodal planes along the *z*-axis. When the height and width of the nano-rod antenna are 280 and 178 nm, the TE_01_ and TE_11_ modes have resonance wavelengths of 680 and 330 nm, respectively. Similarly, the profiles of the TM_01_ and TM_11_ modes are individually extracted at wavelengths of 880 and 330 nm, respectively. We note that the TM_01_ and TE_01_ modes can be widely tuned across the entire visible wavelength range and thus generate the vivid structural colour as observed in the dark-field microscope measurement. The scattered field profile of the TM_01_ mode is less biased to the vertical direction than that of the TE_01_ mode. This can make the scattering properties of the TM_01_ mode less sensitive to the illumination angle.

## Discussion

In summary, we systematically characterized resonant light scattering from a single EBID dielectric optical nano-rod antenna, which covers the whole visible wavelength range for the TE and TM polarizations, depending on the size of the nano-rod. Both dark-field scattering measurements and FDTD simulations confirmed a multicolour optical response of the nano-rod antenna. Our deep understanding of leaky-mode resonances in the EBID dielectric nano-rod will be useful for near-field measurement or for the implementation of new nanophotonic devices. In particular, we expect that EBID nano-structures can function as efficient dielectric antennas on non-planar surfaces such as NSOM tips, AFM cantilevers, or optical fibres.

## Methods

### EBID fabrication of nano-rods

The EBID carbonaceous dielectric nano-rod antennas were formed using vaporized diffusion pump oil molecules in the SEM (Hitachi S-2500) chamber as precursor molecules. The oil used in our diffusion pump is octadecyl-naphthalene. The ambient pressure of the SEM chamber was 3.0 × 10^−5^ Torr. We scanned a 130-pA-current focused electron beam with an acceleration voltage of 30 keV, in the line image mode of ×35000 magnification. The height of the nano-rod can be controlled by the number of electron-beam scans.

### Numerical simulations

To model the EBID carbonaceous material, we used the CP dielectric function that fits the experimental data in Fig. 1d. The dispersive dielectric constant of the silicon substrate was obtained using the dispersion function consisting of the Drude and Lorentz terms in the wavelength range between 300 and 2,000 nm^39^. The cross-sectional morphology of the nano-rod is set to be an arch consisting of an isosceles trapezium and a half circle on top. The base angle of the isosceles trapezium is 75° and the full-width at half maximum is used as the width of the nano-rod. The radius of the half circle is 70 nm. In Fig. 3c,d, the numerical aperture of the microscope objective was considered for a fair comparison with experimental results. The Fourier transformation of the near-field in the position of 600 nm above the substrate along the *x*-axis was performed to obtain the far-field scattering radiation from the nano-rod antenna^40^. Then, the far-field scattering radiation intensity was accumulated within a collection angle of ±25°, corresponding to the numerical aperture of the microscope objective, and was normalized by the incident wave intensity. In Fig. 4a,b, we calculated the total scattering cross-section into the air and substrate and normalized it with the nano-rod height. The time-averaged scattering Poynting vectors from TFSF method are integrated over a box that entirely encloses the nano-rod antenna.

### AFM measurement

To define the height of the fabricated nano-rod antennas, we used AFM (Witec alpha 300s) in the non-contact mode. The AFM tip was operated at a resonance of 250 kHz with a set-point of 0.7 V. We collected statistics of the top surface data along the rod length to obtain the surface roughness.

## Author Contributions

M.-K.S. and H.-G.P. conceived the idea. E.-K.L., J.-H.S., and J.-H.K. fabricated the samples. E.-K.L., J.-H.S., and K.-Y.J. characterized the samples. E.-K.L. and J.-H.S. performed FDTD simulations. All authors discussed the results and wrote the paper.

## Additional Information

**How to cite this article**: Lee, E.-K. *et al.* Resonant light scattering from a single dielectric nano-antenna formed by electron beam-induced deposition. *Sci. Rep.* **5**, 10400; doi: 10.1038/srep10400 (2015).

## Supplementary Material

Supporting Information
