# Supplementary material for: Resonant light scattering from a single dielectric nano-antenna formed by electron beam-induced deposition
Source: Sci Rep. 2015 May 19;5:10400. doi: 10.1038/srep10400 (PMC4437380; doi:10.1038/srep10400)
Supplement: Supporting Information [file srep10400-s1.pdf]

# Supplementary information

# Resonant light scattering from a single dielectric nano-antenna formed by electron beam-induced deposition

Eun-Khwang Lee[1†], Jung-Hwan Song[1†], Kwang-Yong Jeong[2], Ju-Hyung Kang[2],

Hong-Gyu Park[2*], and Min-Kyo Seo[1*]

[1]Department of Physics and Institute for the NanoCentury, KAIST, Daejeon 305-701, Republic of Korea,

[2]Department of Physics, Korea University, Seoul 136-701, Republic of Korea

[†]These authors contributed equally to this work.

*Corresponding Author: hgpark@korea.ac.kr and minkyo_seo@kaist.ac.kr

---

The supplementary information contains text, 4 figures, and 1 table in support of the main body of the text. It discusses four specific topics:

1. Composition of EBID dielectric nano-structures
2. Optical constants of EBID dielectric nano-structures
3. Cross-section morphology of EBID nano-rod antenna in simulation
4. Absorption and extinction cross-section efficiencies of EBID nano-rod antennas

---

# 1. Composition of EBID dielectric nano-structures

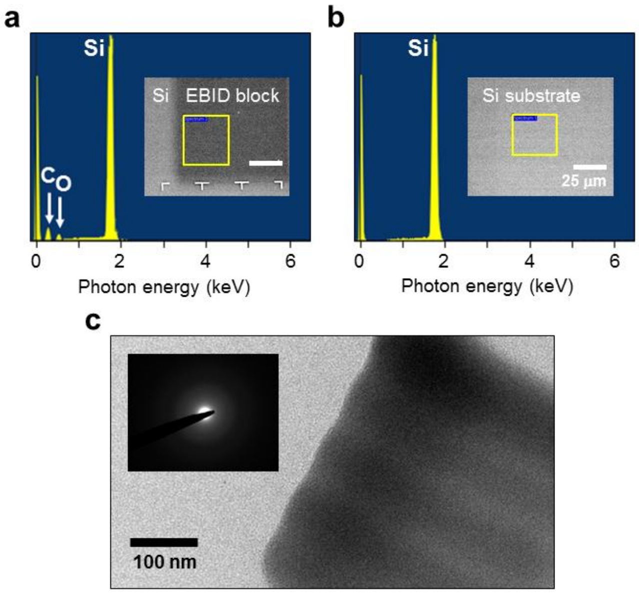


**Figure S1 | Energy dispersive x-ray spectroscopy of EBID block.** (a,b) EDS spectra of an EBID block (a) and bare Si substrate (b). The insets show scanning electron microscope (SEM) images of the EBID block and the bare Si substrate, and the yellow boxes indicate the area for the EDS analysis. (c) TEM image and diffraction pattern of an EBID block.

To characterize the EBID carbonaceous material, we performed energy dispersive x-ray spectroscopy (EDS) measurement. We fabricated an EBID block with a size of 73 × 78 $\mu m^2$, which was fabricated using the same EBID procedure at a current of ~130 pA as the nano-rod antenna (see Method in the main manuscript). In the EDS analysis, 15-keV-electron beam was scanned over the yellow rectangular area on the EBID block and the bare Si substrate as shown in Figures S1a and S1b. On the EBID block, the characteristic spectral lines of C and O are detected at ~0.3 and ~0.6 keV, respectively (Figure S1a). The peak at ~1.8 keV originates from the Si substrate. On the bare Si substrate, the peaks of C and O are not observed at all. The spectrum analysis shows that the EBID block consists of C and O with an atomic-ratio of 88:12. Figure S1c shows a transmission electron microscope (TEM) image and diffraction pattern of an EBID block deposited on a TEM grid. The amorphous characteristics of the EBID structure are clearly observed. We also examined the EBID block fabricated by an electron beam current of ~830 pA, and it shows an atomic-ratio of 88:12 identical to the block fabricated by a current of ~130 pA.

## 2. Optical constants of EBID dielectric nano-structures

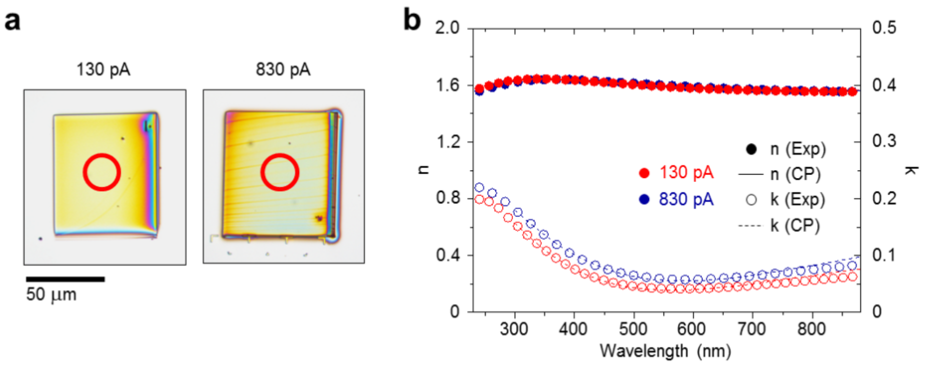


**Figure S2 | Spectroscopic ellipsometry measurement of EBID blocks.** (a) Optical microscopic images of EBID blocks deposited with different electron beam currents of ~130 pA (left panel) and 830 pA (right panel), respectively. The red circle indicates the illumination beam spot for spectroscopic ellipsometry measurement, of which the diameter is ~35 μm. (b) Refractive index (solid circle) and extinction coefficient (hollow circle) spectra measured by the spectroscopic ellipsometry. The solid and dashed lines are the fitted refractive index and extinction coefficient with the CP model, respectively.

We measured the refractive index and extinction coefficient of the EBID carbonaceous material depending on the wavelength using the spectroscopic ellipsometry (Figure S2). Since the nano-rod is too small to obtain the refractive index directly, we analyzed the reflectivity spectrum of the EBID blocks in Figure S2a. The thicknesses of the EBID blocks deposited with different electron beam currents of ~130 and ~830 pA are measured to be ~173 and ~171 nm, respectively, by AFM. In the spectroscopic ellipsometry, the incident beam is obliquely injected with an angle of ~72°. To achieve a high accuracy of the measurement, the flat area on the EBID block was illuminated with a beam spot diameter of ~35 μm (the red circle in Figure S2a). As shown in Figure S2b, the refractive indices of both blocks are almost identical and thus material properties of the EBID structures are insensitive to the EBID fabrication conditions.

We fitted the measured spectra of the refractive index and extinction coefficient to the critical points (CP) model with two critical point pairs:

$$\varepsilon(\omega) = \varepsilon_\infty + A_1\omega_1\left(\frac{e^{i\phi_1}}{\omega_1-\omega-i\gamma_1}+\frac{e^{-i\phi_1}}{\omega_1+\omega+i\gamma_1}\right) + A_2\omega_2\left(\frac{e^{i\phi_2}}{\omega_2-\omega-i\gamma_2}+\frac{e^{-i\phi_2}}{\omega_2+\omega+i\gamma_2}\right).$$

Here, $\varepsilon_\infty$ is the background dielectric constant, and $\omega_i$, $\phi_i$, $\gamma_i$, and $A_i$ are the angular frequency, phase angle, collision frequency, and amplitude of the $i^{th}$ oscillator, respectively. Table S1 shows the fitting parameters for the EBID carbonaceous material deposited by an electron beam current of ~130 pA, which was employed to form the nano-rod antennas. The dispersive refractive indices were handled in the finite-difference time-domain (FDTD) simulations by the auxiliary differential equation (ADE) method.

**Table S1.** Parameters of the CP model for EBID carbonaceous material

| Parameters | Values |
|---|---|
| Background dielectric constant, $\varepsilon_\infty$ | 1.5 |
| Angular frequency of oscillator 1, $\omega_1$ ($s^{-1}$) | $4.9 \times 10^{15}$ |
| Collision frequency of oscillator 1, $\gamma_1$ ($s^{-1}$) | $5.5 \times 10^{15}$ |
| Amplitude of oscillator 1, $A_1$ | -1.3 |
| Phase angle of oscillator 1, $\phi_1$ (rad) | 1.6 |
| Angular frequency of oscillator 2, $\omega_2$ ($s^{-1}$) | $6.8 \times 10^{14}$ |
| Collision frequency of oscillator 2, $\gamma_2$ ($s^{-1}$) | $2.4 \times 10^{15}$ |
| Amplitude of oscillator 2, $A_2$ | 7.3 |
| Phase angle of oscillator 2, $\phi_2$ (rad) | 0.25 |

## 3. Cross-section morphology of EBID nano-rod antenna in simulation

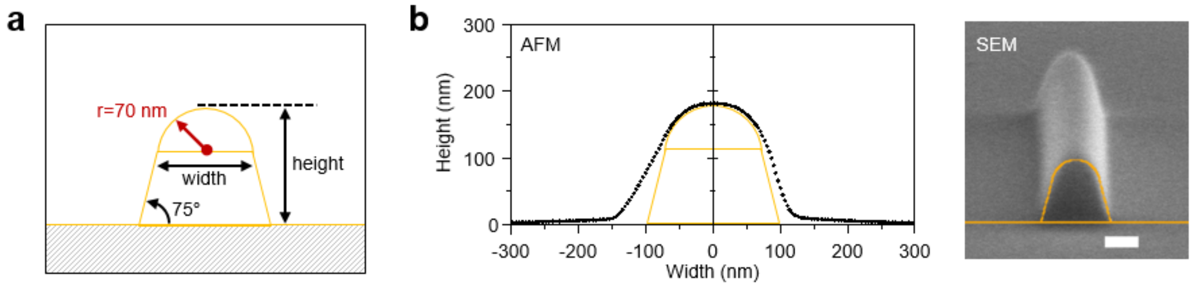


**Figure S3 | Cross-section morphology of EBID nano-rod antenna.** (a) Scheme of the simulated morphology of EBID nano-rod antenna. (b) AFM (left) and SEM (right) measurements of an EBID nano-rod antenna with a height of ~180 nm. In the SEM image, the scale bar is 100 nm. The contour line of the simulated morphology (orange line) is overlaid on the AFM and SEM images.

To reflect the cross-section morphology of the actually fabricated nano-rods, we used an isosceles trapezium in combination with a half circle on top (Figure S3a). The base angle of the isosceles trapezium is 75$^{o}$. The full-width at half maximum is used as the width of the nano-rod, which varies linearly depending on the height. The radius of the half circle is 70 nm. In Figure S3b, the orange coloured contour line indicates the cross-section morphology of the nano-rod used in simulation.

## 4. Absorption and extinction cross-section efficiencies of EBID nano-rod antennas

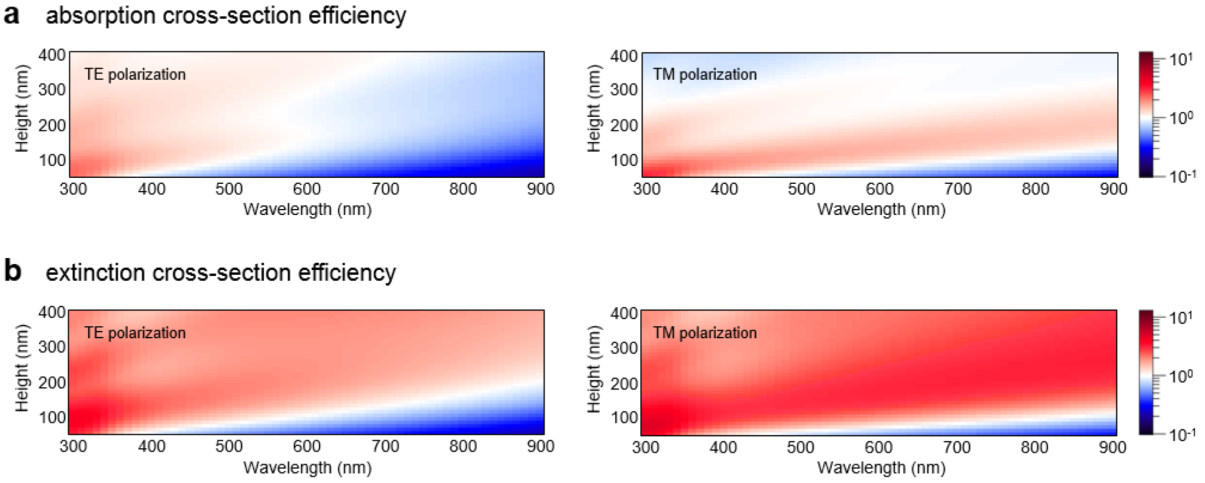


**Figure S4 | Calculated absorption and extinction cross-section efficiencies.** (a) 2D maps of the nano-rod absorption cross-section efficiency on a log scale as a function of the wavelength and the height of the nano-rod antenna for TE- (left panel) and TM-polarized (right panel) illumination. (b) 2D maps of the extinction cross-section efficiency for TE- (left panel) and TM-polarized (right panel) illumination.

For a better understanding of the resonant absorption and scattering by the EBID nano-rod antenna, we calculated 2D maps of the absorption and extinction scattering efficiencies as a function of the incident wavelength and height of the nano-rod antenna. The absorption efficiency is defined as the ratio of the absorption cross-section to the height of the nano-rod antenna, and the extinction efficiency is the sum of the absorption and scattering efficiencies. The absorption cross-section is obtained by calculating the absorbed power flux to the EBID nano-rod and normalizing it to the incident power. The absorption and extinction efficiencies show the resonance features similar to the scattering efficiency of Figure 4a-b in the main manuscript. Due to a relatively high extinction coefficient of the deposited material, the absorption efficiency is non-negligible in the short wavelength region and shows a tendency analogous to the measured imaginary refractive index in Figure S2b. At the $TM_{01}$ mode resonance of the nano-rod antenna with a height of 280 nm ($\lambda$=880 nm), the absorption and extinction efficiencies are ~1.13 and ~3.09, respectively. At the $TE_{01}$ mode resonance ($\lambda$=680 nm), the absorption and extinction efficiencies are ~0.87 and ~1.78, respectively.
